# Extracellular Vesicles as Biomarkers Carriers in Bladder Cancer: Diagnosis, Surveillance, and Treatment

**DOI:** 10.3390/ijms22052744

**Published:** 2021-03-09

**Authors:** Natalia Georgantzoglou, Alexandros Pergaris, Christos Masaoutis, Stamatios Theocharis

**Affiliations:** First Department of Pathology, Medical School, National and Kapodistrian University of Athens, 75, M. Asias Str., Bld 10, Goudi, GR11527 Athens, Greece; natalia.georgantzoglou@gmail.com (N.G.); alexperg@yahoo.com (A.P.); cmasaout@med.uoa.gr (C.M.)

**Keywords:** extracellular vesicles, exosomes, biomarkers, bladder cancer, diagnosis, prognosis, treatment

## Abstract

Exosomes are extracellular vesicles, enriched in biomolecular cargo consisting of nucleic acids, proteins, and lipids, which take part in intercellular communication and play a crucial role in both physiologic functions and oncogenesis. Bladder cancer is the most common urinary malignancy and its incidence is steadily rising in developed countries. Despite the high five-year survival in patients diagnosed at early disease stage, survival substantially drops in patients with muscle-invasive or metastatic disease. Therefore, early detection of primary disease as well as recurrence is of paramount importance. The role that exosomal biomarkers could play in bladder cancer patient diagnosis and surveillance, as well as their potential therapeutic applications, has not been extensively studied in this malignancy. In the present review, we summarize all relevant data obtained so far from cell lines, animal models, and patient biofluids and tissues. Current literature suggests that urine is a rich source of extracellular vesicle-derived biomarkers, compared with blood and bladder tissue samples, with potential applications in bladder cancer management. Further studies improving sample collection procedures and optimizing purification and analytical methods should augment bladder cancer diagnostic, prognostic, and therapeutic input of extracellular vesicles biomarkers in the future.

## 1. Introduction

Bladder Cancer (BC) is the ninth most common malignancy worldwide and the most common genitourinary malignancy in both men and women, with incidence being over four times higher in men. Histopathologically, BC can broadly be divided in urothelial and non-urothelial BC, with squamous cell carcinoma (SCC) being the most common among the latter. Urothelial carcinoma accounts for >90% of BC in Europe and North America, while SCC is the predominant type in Middle Eastern Regions where *Schistosoma Haematobium* is endemic [1,2,3].

Almost 80% of BCs present as non-muscle invasive disease. Among those 60% are confined to the bladder mucosa (pTa), 30% invade the submucosa (pT1) and 10% present as Carcinoma in Situ. The five-year survival for BC correlates with disease stage at diagnosis and is as high as 95.8% for carcinoma in situ (Tis)and as low as 4.6% for metastatic disease, a fact which highlights that accurate and timely diagnosis is of paramount importance for the prognosis of BC patients [4].

Cystoscopy and cytology are the two main modalities that are currently used for BC diagnosis [5]. Cytology has a relatively low sensitivity, which was estimated around 34% in a meta-analysis by Lotan et al. [6]. Sensitivity was lower for low grade tumors, which could be attributed to low grade tumors having fewer cell to cell adhesion disruptions and thus less exfoliating capacity. Cystoscopy is the current gold standard for BC diagnosis, owing to its overall higher sensitivity for BC detection comparing to other methods, however, the detection of in situ carcinoma remains a challenge [7]. Recent advances in fluorescent light cystoscopy have yielded promising results as photodynamic cystoscopy is reported to have over 90% sensitivity (comparing to 62% of white light cystoscopy) in detecting carcinoma in situ [8]. Cystoscopy, however, is an invasive procedure and it has been associated with urethral stricture, perforation, hemorrhage and infection [9].

The need for non-invasive tools for diagnosis and follow up led to the identification of protein biomarkers in the urine, such as Nuclear Matrix Protein 22 (NMP-22) and Bladder Tumor Antigen, which are currently FDA approved but are yet to be widely adopted by the urologic community [10]. Nevertheless, despite their higher sensitivity comparing to cytology, they are not adequately sensitive in order to eliminate the need for cystoscopy. In addition, similarly to cytology, they lack sensitivity for low grade BCs and the positive predictive value can be adversely affected by the presence of hematuria, calculi, benign genitourinary disease, and inflammation [11].

The abovementioned shortcomings of the current diagnostic modalities underscore the importance of extensive research in order to identify additional sensitive and specific biomarkers, that could be of great value in the early detection of BC as well as in surveillance of patients already diagnosed with the disease. The latter is of paramount importance as BC is associated with high recurrence rates and an estimated 10% of patients with Ta and T1 disease eventually progress to muscle invasive disease, while the risk has been reported to be almost five times higher for patient with in situ carcinoma [12].

Over the precedent years, extracellular vesicles (EVs) have attracted an increasing interest in oncology and are bound to find clinical applications in the proximate future. They constitute a heterogeneous group of cell-secreted membrane-bound vesicles containing a rich biomolecular cargo of nucleic acids, lipids and proteins [13]. Their implications for cancer biology and treatment are of particular interest: neoplastic cell-derived EVs deliver oncoproteins/oncopeptides and various nucleic acid species capable of inducing changes in the tumoral microenvironment, including premetastatic niche formation [13,14,15,16], provide a rich source of potential novel biomarkers for liquid biopsy allowing for the detection of oncogenic mutations and capturing clonal heterogeneity, and could be used in cancer vaccine development [17]. EVs are also being studied as drug delivery vectors: small chemotherapeutic drug molecules, siRNAs, anti-miRNAs, mRNAs, and proteins have been successfully packed into EVs [13,14,15,16]. Inherent characteristics of the EVs, such as their low immunogenicity and little accumulation in the tissues in the long term, could potentially provide a safe, non-toxic alternative to the current standard of treatment [15].

In this review, we aim to present the current knowledge base on the potential applications of EVs biomarkers in clinical diagnostics and surveillance of patients with BC, as well as their prospective therapeutic implementations.

## 2. Biology and Function of EVs

EVs possess wide-reaching bio-signaling properties, generally in the form of intercellular exchange of cellular components. They are broadly divided into two main categories, namely exosomes and microvesicles, which differ in terms of biogenesis. Exosomes on the one hand usually measure 30–100 nm in diameter and are generated within the endosomal system as follows: the inward budding of the multivesicular endosome (MVE) membrane forms intraluminal vesicles (ILVs), which are later secreted as exosomes upon fusion of the MVE membrane with the plasma membrane. Microvesicles on the other hand usually measure 50–1000 nm (although tumor-derived ones may reach up to 10 μm in diameter) and are formed by an outward blebbing and subsequent fission of the cell membrane. Specialized sorting machineries of the cell are responsible for packaging various biomolecules in EVs and achieve this by clustering them in membrane microdomains on the internal surface of the forming EV. EVs carry sets of cell-type-specific proteins depending on their cell of origin. One cell type may also produce distinct EV subpopulations: polarized epithelial cells, for example, shed exosomes of different composition from the apical and basolateral part. Therefore, sub-species within the two broad EV categories can be recognized. When an EV reaches the target cell, it may remain bound to the cellular surface activating intracellular signaling pathways, be internalized by phagocytosis of micropinocytosis, or fuse with the plasma membrane, directly releasing its contents into the cytoplasm.

The overlap in size and composition of exosomes and microvesicles renders the distinction between the two, especially in terms of isolation, a technical challenge [16,17]. Protein aggregates, lipoparticles, viruses, and cell debris must be removed to achieve EV purification. Isolation techniques include differential ultracentrifugation, filtration-based procedures, flotation on density gradients, size exclusion chromatography, and immunoaffinity-based separation or combinations thereof [11]. EV isolation methods are continuously evolving and getting standardized, leading to better reproducibility and comparability of results across different studies. A deeper understanding of the biology and mechanisms behind biomolecule sorting and packaging in EVs could play a crucial role in the optimization of isolation methods in the future [18].

EVs are implicated in both normal physiological and pathological processes. EVs take part-among others-in cell migration, such as neutrophil chemotaxis, remodeling of the extracellular matrix, immunity, and inflammation (antigen presentation, T-cell-derived signaling, or dendritic-to-dendritic-cell communication), morphogenesis, and CNS physiology (extrasynaptic control of neuronal communication and glia-neuron interaction) [19]. In addition to their normal functions, they are also implicated in the pathology of a wide spectrum of human disease, including cancer, Parkinson’s disease, Alzheimer’s disease, peripartum cardiomyopathy, coronary artery disease, and various types of nephropathies [20].

## 3. EVs in Bladder Cancer Pathogenesis

There is accumulating evidence that EVs play a pivotal role in tumorigenesis by increasing invasiveness and migration, enhancing angiogenesis, promoting the activation of cancer associated fibroblasts and augmenting cancer cell proliferation [21,22]. However, research data regarding the role of EVs in BC pathogenesis remain currently limited.

Ogorevc et al. first reported vesicular communication between high grade BC cell line T24 and urinary papilloma cells in vitro. Using DiO dye and electron microscopy, they observed increased membrane budding in DiO-stained malignant cells and consequently identified the presence of DiO fluorescence in papilloma cells, after the latter were incubated with the cancer cell line derived EVs [23].

Wu et al. investigated the role of EVs in bladder tumorigenesis both in vitro and in vivo, using EVs purified from high grade carcinoma cell lines TCCSUP and T24 via ultracentrifugation. More specifically, human urothelial cells treated with TCCSUP-derived EVs for 13 weeks, displayed increased proliferation capacity and induced tumor formation when engrafted in athymic mice. Subsequently, stress related gene alterations and DNA damage such as upregulation of anti-oxidative stress genes and increased double strand breaks were identified with quantitative PCR. In addition, N cadherin, which is known to enhance invasiveness, was found to be upregulated and E-cadherin, which has the opposite effect, downregulated, in agreement with data reported in other cancer types [24,25,26]. Finally, it was demonstrated that EVs (exosome) treated cells harbored increased unfolded protein response of the endoplasmic reticulum, which is characteristic of the stress imposed on the ER by increased cell proliferation [27].

Similar results regarding decreased E-cadherin expression and increased invasiveness and migration were reported by Franzen et al., who treated urothelial cells with EVs (exosomes) derived from muscle invasive BC cells (UMUC3 and T24 cell lines). The urothelial cells demonstrated increased amoeboid migration when plated in the presence of muscle invasive bladder cancer (MIBC)-derived EVs on collagen IV coated glass. Additionally, MIBC exosomes induced epithelial to mesenchymal transition (EMT) and upregulation of mesenchymal markers expression, such as vimentin and snail. EMT is a process during which cells lose their polarity and cell to cell adhesions and has been implicated in the pathogenesis of many different cancer types [28,29]. Indeed, increased expression of mesenchymal markers is associated with higher grade and stage in BC. EV-induced migration and mesenchymal markers upregulation in the primary urothelial cells were reproduced when BC patient urine and barbotage urine was used. Interestingly, increased expression of mesenchymal markers was more pronounced in the barbotage samples [30]. The induction of EMT by BC derived EVs (exosomes) was also demonstrated by Goulet et al.: healthy fibroblasts co-cultured with bladder cancer derived-EVs acquired features of cancer associated fibroblasts (CAFs), i.e., spindle like morphology and increased SMA expression. When the cell medium from the induced CAFs was used to grow RT4 BC cells the latter eventually exhibited EMT phenotype characteristics, such as decreased E-cadherin and increased N-cadherin and vimentin expression levels [31].

Additional tumorigenic properties of BC EVs were further elucidated by Yang et al., who demonstrated upregulation of Bcl2 and Cyclin D, downregulation of the pro-apoptotic Bax and Caspase-3, as also a dose dependent inhibition of apoptosis in BC cell lines T24 and 5637, following treatment with BC derived EVs (exosomes) [32].

**Figure 1:**
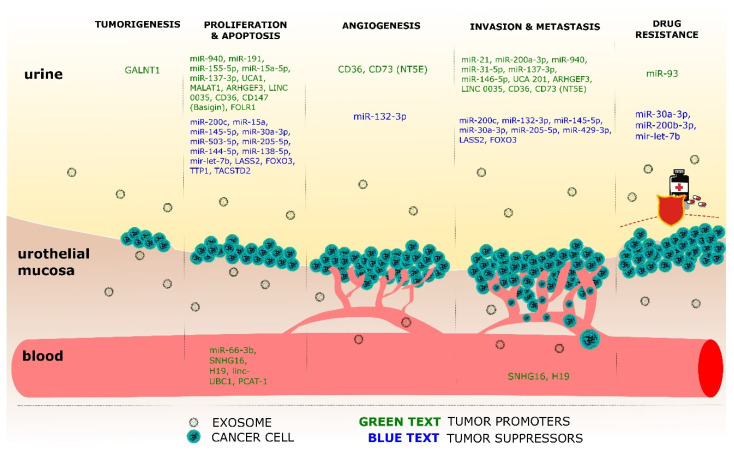
Extracellular vesicles (EV) biomarker isolation in urine and blood and their role in different stages of bladder cancer (BC) tumorigenesis.

To sum up, preclinical data suggest that EVs promote BC oncogenesis and progression by affecting the cell cycle, promoting EMT, and shaping tumoral stroma. The role of EVs-derived macromolecules in the various stages of BC tumorigenesis and the biofluid in which they have been isolated in the existing literature, is summarized in Figure 1.

## 4. EVs as Biomarker Carriers for Bladder Cancer Diagnosis

In the precedent years EVs have captured the interest of the scientific community, as their properties render them attractive targets in clinical diagnostics [33,34]. They contain a number of macromolecules, such as proteins and nucleic acids, that have been implicated in a wide array of human disease apart from cancer, such as Alzheimer’s disease, Parkinson’s disease Hepatitis C and Prion disease [35]. Additionally, they can be isolated from human biofluids, such as urine, saliva, amniotic fluid, and blood. It should be noted, however, that EVs isolation is a tedious process and its technical standardization remains to be determined [36,37], a factor that has undoubtedly hindered their wider application in diagnostics so far.

### 4.1. Protein Biomarkers

EVs (exosomes) are enriched in transmembrane and cytosolic proteins, some of which are considered common exosomal markers regardless of the cell of origin. It is well established in the literature that exosomal membranes are rich in members of the tetraspanin family, namely CD63, CD81, CD9, as well as ESCRT proteins, including HSPs and TSG101 [38].

In concordance with these data, Welton et al., using Western blotting and flow cytometry in exosomes purified from HT1376 BC cells, reported strong expression of CD9 as well as significant expression of CD81 and CD63. The same group use Liquid Chromatography (MALDI-TOF/TOF-MS) and identified 353 proteins in BC derived exosomes, including members of ESCRT family, HSP (hsp70, hsp90), cytoskeletal elements (actin, myosin, and cytokeratins), and a wide array of transmembrane proteins (integrins, EGF-R, CD44, mucin-1, and syndecan.) In an effort, to eliminate any non-exosomal contaminant effect, ultracentrifugation followed by Western blot and flow cytometry was used, and the presence of 18 proteins, including β1 integrin, a6 integrin, CD36, CD73, CD10, MUC1, basigin, and 5T4, was validated. The abovementioned method was consequently employed in order to purify exosomes from patients’ (*n* = 7, 3 BC patients, 4 controls) urine and differential expression levels of CD36, CD44,5T4, basigin, and CD73 between cancer patient and controls were noted [39].

Proteomic analysis using spectrometry conducted by Chen et al., between BC patients and hernia control patients, demonstrated 80% overlap with the protein molecules identified by Welton and colleagues. TACSTD2, a calcium signal transducer implicated in the pathogenesis of several other tumors [40,41], was a newly identified potential biomarker, as it was almost exclusively expressed by cancer cells [42].

Lin et al. used MALDI-TOF spectrometry in urine exosomes analysis of BC patients and healthy controls. The common exosomal markers Alix and TSG101 were used in Western blotting to confirm successful exosome isolation from the samples. The results revealed statistically significant overexpression of α1 antitrypsin and H2B1K, a histone involved in gene expression control and DNA damage response, in urothelial cancer compared to noncancerous tissues. These findings were verified by immunohistochemistry. The sensitivity and specificity of the combination of the two proteins in detecting BC was estimated 62.7% and 87.59% respectively, and interestingly, the diagnostic accuracy of these markers was higher than the traditionally use occult blood test. It was also demonstrated that increased α1 antitrypsin and H2B1K expression was associated with higher grade tumors [43].

Yazarlou et al. assessed the differential expression of seven cancer testis antigens and NMP22 between EVs (exosomes) derived from the urine of patients with BC, healthy controls and patients with benign urologic disease (Benign Prostate Hyperplasia; BPH, obstructive uropathy and bladder calculi). Results revealed that MAGE-B4 was upregulated in BC patients comparing to healthy controls and exosomal NMP 22 was higher in BC samples comparing to BPH [44].

### 4.2. MicroRNAs and IncRNAs

MicroRNAs (MiRs) are small non-coding RNAs, consisting of 21–25 nucleotides, that regulate gene expression by repressing translation or promoting mRNA degradation [45]. EVs (exosomal) miRNA taken up by recipient cells can modulate their gene expression and, therefore, their function. In a pilot study, Perez et al. profiled the exosomal RNA from 5 UBC patients and 6 non-cancer controls, finding that the EVs of healthy samples contained a higher number of transcripts compared to their cancer counterparts. Of the genes expressed, cancer exosomes consistently contained *GALNT1* and *LASS2* transcripts while lacking *FOXO3* and *ARHGEF39* expression, with the opposite being true for the healthy samples. The small sample size of this study, however, warrants further research in order to elucidate the potential significance of these genes [46].

Matsuzaki et al. conducted urinary EVs (exosomal) miRs analysis in patients with both invasive and noninvasive disease vs healthy controls and identified five miRs (miR155-5p, miR15a-5p, miR21-5p, miR132-3p, and miR31-5p). Among these, miR21-5p was identified as a potential biomarker for BC diagnosis due to the estimated considerable sensitivity (75%) and specificity (98%). Notably, miR-21-5p was increased in samples from patients, with false negative cytology results, which renders it a potent biomarker for early disease diagnosis [47].

According to a miRs analysis of cell-free urine from BC patients with various pathologic stages (TaG1, T1G3, >T2, and CIS) and healthy controls, seven miRs (miR-200c, miR-93, miR-940, miRlet7b, miR-191, miR-21, and miR-15a) were significantly increased in the urine from patients with urothelial cancer and their levels correlated with disease stage, with the highest levels noted in patients with >T2 and T1G3 disease. This underscores the potential of exosomal miRs as disease progression biomarkers [48].

MiR-66-3b was identified as a potential diagnostic biomarker in a study by Yin et al., who isolated EVs (exosomes) from the plasma of BC patients and healthy controls, using the common exosomal markers CD9 and CD81. MiR-66-3b levels were significantly increased in BC patients compared to healthy controls. MiR66-3b overexpression was associated with upregulation of vimentin and downregulation of E-Cadherin, characteristics of the EMT phenotype, and inhibited the expression of ERF, which is known to regulate proliferation-related genes [49].

Apart from the miRs, long non-coding RNAs (lncRNAs) have also been associated with genomic alterations in common malignancies, including prostate and colon cancer [50,51]. Inc RNAs consist of >200 nucleotides and are implicated in a wide array of cellular functions, including chromatin remodeling, dosage compensation, and genomic imprinting [52,53,54]. In a large series that included urinary samples of 59 transitional cell carcinoma (TCC) patients and 49 non-cancerous controls, Yazarlou et al. investigated the role of specific EVs (exosomal) lncRNAs in BC diagnosis. LINC0035, UCA1-203, and MALAT1 lncRNA expression was significantly higher in TCC patients, while UCA-201 expression was decreased [55]. Similarly, Zhang et al. explored the diagnostic potential of exosomal lncRNAs in serum samples of 100 BC patients and an equal number of controls, and validated their results in a larger independent cohort. It was demonstrated that three lncRNAs, PCAT-1, UBC-1, and SNH G16, were significantly increased in the serum of BC patients comparing to controls, and their diagnostic accuracy was markedly higher than the estimated accuracy of urine cytology [56].

## 5. EVs as Biomarkers Carriers for Bladder Cancer Disease Progression and Recurrence

Non-muscle invasive BC (NMIBC) is characterized by high recurrence rates, as the one-year recurrence rate is 15–61%, and the five-year recurrence rate can be up to 78%. Among the patients who recur, almost 40% will do so without progression, while approximately 35% will progress to muscle-invasive disease. Furthermore, among those who progress, around 30% will die of BC. These statistics underscore the need of developing sensitive diagnostic tools for intensive surveillance of patients diagnosed with and treated for BC [57].

A recent study demonstrating differential EVs (exosomal) miRs expression between NMIBC and MIBC highlights the potential role of EVs biomarkers in BC patients’ surveillance [58]. The authors used microarray analysis and qRT-PCR on tissue and urinary samples to show that MIBC is characterized by a specific miRNA profile: miR146b-5p and miR155-5p were upregulated while miR138-5p, miR144-5p, and miR200a-3p were significantly downregulated. In tissue samples, four out of the five miRs (all except miR144-5p) were differentially expressed between pTa and ≥ pT2 tumors. In the urine samples, pTa tumors could be differentiated from pT2 tumors based on miRs expression but not from pT3-4 tumors. Among lncRNAs, UBC1, and SNHG16 were significantly increased in patients with MIBC comparing to NMBIC. Additionally, higher UBC1 expression correlated with decreased recurrence-free survival in NMBIC patients, independently of tumor stage.

Increased serum EVs (exosomal) IncRNAH19 in BC patients was associated with poorer prognosis, while multivariate regression analysis showed that H19 levels and TNM stage were independent prognostic factors for overall survival [59]. High urinary exosomal MALAT-1 and PCAT-1 levels were correlated with decreased recurrence free survival in NIMBC patients [60].

The high recurrence rates of BC in patients that have already undergone cystectomy, highlight the significance of undetected cancer cells and micro-metastases in patient prognosis [61]. Hiltbrunner et al. attempted to trace cancer cell-derived exosomes in completely downstaged BC patients who had undergone cystectomy, using urinary samples from the bladder and the ureter. It was demonstrated that the isolated exosomes harbored an altered proteomic profile, which was associated with cancer metabolism. Specifically, the exosomes were enriched in proteins that take part in gluconeogenesis, glycolysis, pentose phosphate pathway, and glutathione metabolism, pathways crucial for the viability and proliferation of cancer cells. The authors also identified potential protein biomarkers (TPP1, TMPRSS2, and FOLR1) that could be utilized as markers for disease recurrence and prognosis [62].

There is also evidence that certain miRs signatures are associated with enhanced metastatic potential of BC cells. Ostenfeld et al. reported that exosome-dependent release of miR-23b is associated with altered metastatic dynamics. MiR-23b harbors anti metastatic properties, such as inhibition of angiogenesis and invasion, and its disposal is therefore associated with poor prognosis [63].

## 6. EVs as Potential Therapeutic Vectors

The current therapeutic standard for BC encompasses a multimodal approach that largely depends on the disease stage. For noninvasive BC, transurethral tumor resection and a single post -TUR intravesical chemotherapy instillation is performed in all patients, while patients with intermediate or high-risk tumors also require BCG induction and/or induction maintenance chemotherapy [64]. For non- metastatic MIBC, cystectomy and pelvic lymph node dissection, along with neo adjuvant cisplatin-based chemotherapy, is the standard of treatment. Platin-based chemotherapy is also the cornerstone of therapy for metastatic disease, while this subset of patients can benefit from offered targeted therapies and radiation [65,66].

Several of the EVs physical properties have rendered them a promising potential therapeutic vector for cancer in the recent years. Their small size, permeability owing to the lipid bilayer, stability, low immunogenicity, and capacity of transferring biomolecules, such as RNA and proteins, make the exosomes attractive chemotherapeutic drug carriers [67,68,69]. Recently, Yong et al. demonstrated that doxorubicin-loaded, exosome-based nanoparticle injection in mice with subcutaneous tumors resulted in significant tumor volume reduction and prolonged survival [70].

Currently, available data regarding exosome utilization in BC treatment are limited. Cai et al. examined the effect of exosomal miR-133b on BC cell proliferation both in vitro and in vivo [71]. The authors isolated exosomes from BC tissue and serum from BC patients, as well as exosomes from BC cell lines (T24 and 5637), reversely transcribed RNA into cDNA and subsequently performed RT-PCR using miR133b specific primer. Cells that had been transfected with the miR133b mimics were then co-cultured with BC cells and cancer cell proliferation was assessed. Tumor proliferation was also evaluated in mice, in which T24 induced tumors were injected with miR133b–mimic-loaded exosomes. The authors concluded that cancer cell proliferation was reduced in the presence of miR133b-loaded exosomes, both in vivo and in vitro, and attributed its anti-oncogenic properties to upregulation of Dual Specificity Phosphatase -1 (DUSP-1). DUSP-1 is a negative regulator of MAPKs, and thus plays a critical role in modulating cell proliferation an apoptosis, innate immune responses and autophagy [72,73,74]. They also observed that expression of miR133b was significantly downregulated in BC tissues compared to normal controls.

Similar in vitro and in vivo experiments, by Li et al., on cell lines and BC patient tissue, regarding the role of exosomal miR375-3p in BC suppression, indicated that miR375-3p–loaded exosomes increased cancer cell apoptosis by increasing caspase 1 and caspase 3 expression while on the other hand inhibited cancer cell proliferation and migration. It was demonstrated that the former was mediated through WNT/β catenin pathway inhibition and the latter by downregulation of ICAM, MMP2, and MMP9 expression [75].

The capacity of exosomes to inhibit cancer cell proliferation was also demonstrated in a study using miR29c containing adenovirus to infect BC cell lines (BIU-87). The cells were divided in three groups: cells infected with mir29c adenovirus (Ad-miR), cells infected with adenovirus, and controls. Culture supernatant was collected from the three groups and the exosomes were isolated by ultracentrifugation. Consequently, BIU-87 cells were treated with exosomes from the three different groups and apoptosis rate was measured using flow cytometry. The authors concluded that apoptosis rate of BIU-87 cells treated with exosomes derived from the Ad-miR group was significantly increased compared to the cells treated with exosomes of the other groups. It was also demonstrated that the former expressed decreased levels of pro-survival proteins BCL-2 and MCL-1, thus elucidating the possible mechanism of apoptosis induction [76].

Current data on EVs content biomarkers, involved in diagnosis, prognosis and therapy and their contribution in Bladder cancer pathogenesis are summarized on Table 1, Table 2 and Table 3. Data regarding the target molecules and mechanisms of action of the EV-derived protein biomarkers, miRNAs and lncRNAs, presented in Table 1, Table 2 and Table 3 were acquired from the following databases: miRDB [77], miRbase [78], MarkerDB [79], and lnc2Cancer [80]. 

The abovementioned data highlight the promising role of EVs in modulating the tumor microenvironment in favor of the host, by halting BC tumor growth and invasion, but further research is certainly warranted. The tumor microenvironment could also be manipulated by augmenting the host immune response against cancer cell and, indeed, there are currently several studies underway examining the potential role of EVs in immunotherapy in several types of cancer [81].

## 7. Conclusions

The limitations of the current diagnostic methods for BC render research efforts, for the determination of novel biomarkers, a necessity. In the recent years, EVs (exosomes) derived biomarkers have attracted the interest of the scientific community as a potential candidate for primary disease diagnosis as well as early detection of recurrence, a prevalent phenomenon among BC patients. Existing literature suggests that EVs biomarkers display a higher sensitivity in detecting BC compared to the conventional screening methods, namely, cytology and urine occult blood test, but are not sensitive enough to eliminate the need for cystoscopy with tissue sampling, which remains the cornerstone of BC diagnosis. Thus, EVs biomarkers show promising clinical utility as an adjunct tool, in order to increase the sensitivity of cytology, especially in cases of low grade tumors, in which decreased exfoliating capacity impacts diagnostic accuracy. They could also be used in conjunction with cystoscopy, in instances that cystoscopic examination typically yields suboptimal results, such as in small, flat tumors, or in surveillance examination of patients who have received intravesical BCG. In the latter, erythematous lesions that are oftentimes disregarded as inflammatory in origin could harbor recurrent neoplastic foci. It should be noted, however, that further research should be conducted in order to determine how EV biomarker secretion and isolation is affected by the concurrent inflammation, induced by intravesical chemotherapy. In addition, the isolation of tumor derived EVs in completely downstaged patients, highlights the promising potential role of the EVs (exosomes) as tumor-load markers in patients without clinical, cystoscopic, or imaging findings. The abundance of EV derived biomarkers in BC patient urine is an additional advantage of their potential implementation as diagnostic tools in clinical practice, as urine constitutes an easily accessible biofluid.

However, it should be noted that there are still numerous limitations to overcome in order for the exosomal biomarkers to be implemented in clinical diagnostics. First and foremost, the lack of standardized isolation procedures remains a challenge. In the existing literature, ultracentrifugation seems to be the most commonly used procedure, when it comes to isolating tumor derived EVs (exosomes) from urine samples but often yields impure exosomes due to the abundance of soluble proteins and the intensive nature of the procedure. Further comparative studies in order to determine the optimal isolation method are imperative. Additionally, a uniform protocol regarding urine sample handling should be implemented as urine collection, presence of proteinases and storage conditions can all affect exosome isolation. Finally, despite the promising results of the existing studies, EV (exosomal) biomarkers should be validated in larger samples, including patients of various disease stage, as well as controls with other urologic malignancies, in order to better appreciate the sensitivity and specificity of the proposed biomarkers. Future research endeavors in large patient cohorts could also determine which type of biofluid-urine or serum- is optimal for BC EV biomarker isolation and level measurement. Existing literature suggests that urine is a richer source of biomarker compared to blood, but further research is warranted. Despite all current limitations, we believe that EVs can contribute to shaping a personalized, precision-focused and minimally invasive management of BC in the long term.

## Figures and Tables

**Table 1:** Molecular pathways of EV protein biomarkers in bladder cancer pathogenesis.

Protein Extracellular Vesicle Biomarkers in Bladder Cancer			
Proteins	Effect	Mechanism of Action	Ref.
Promoters of Oncogenesis			
CD36 ^a^	↑ migration, proliferation and angiogenesis	↑ Fatty acid uptake	[37]
5T4 *	↑ EMT and migration	↑ CXCL12/CXCR4 chemotaxis ↓ Normal WNT/β-catenin pathway	[37]
CD44 *	↑ proliferation, migration and angiogenesis	Adhesion molecule Docking of proteases on cell membrane	[37]
CD73 (NT5E) ^a^	↑ angiogenesis, metastases and invasion	Regulates cellular signaling with extracellular matrix components such as fibronectin and laminin	[37]
CD147 (Basigin) ^a^	↑ proliferation	Regulates glycolytic metabolic pathways	[37]
Alpha 1-antitrypsin ^a,^*	↓ apoptosis	Immunity regulation	[41]
MAGEB4 ^a,^*	↑ tumorigenesis and proliferation	↑ ubiquitination and degradation of various tumor suppressors (p53)	[42]
NMP-22	↑ proliferation	Part of nuclear mitotic apparatus	[42]
FOLR1 ^a^	↑ proliferation	↑ folic uptake in tumor cells ↑ nucleic acid synthesis ↑ STAT3 activation	[60]
Suppressors of Oncogenesis			
TACSTD2 ^a,^**	↑ apoptosis	Participates in TAp63-dependent apoptosis	[38,39,40]
H2B1K ^a,^*	↓ tumorigenesis	Regulates response to DNA damage ↑ Transcriptional expression of tumor suppressor genes	[41]
TTP1 ^a^	↑ apoptosis	mitochondrial toxin	[60]

^a^: measured in urine exosomes; *: mechanism described in various cancers; **: mechanism described in squamous cell carcinoma.

**Table 2:** Molecular pathways of EV miRNA biomarkers in bladder cancer pathogenesis.

miRNA Extracellular Vesicle Biomarkers in Bladder Cancer			
miRNAs	Effect	Mechanism of Action	Ref.
Promoters of Oncogenesis			
miR-15a-5p ^a^	↑ proliferation	post transcriptional regulation of MYB oncogene	[45]
miR-31-5p ^a^	↑ migration and invasion	MAGI2-AS3/miR-31-5p/TNS1 axis	[45]
miR-21 ^a^	↑ invasion	↓ AKT and MAPK pathways	[45,46]
miR-155-5p ^a^	↓ apoptosis	↓ TP53INP1 expression	[45,56]
miR-940 ^a^	↑ proliferation, migration and invasion	↑ expression of INPP4A, GSK3b, c-MYC, cyclin D, β-catenin ↓ p-27 expression	[46]
miR-191 ^a^	↓ apoptosis	↓ circ-FOXO-3 expression	[46]
miR-93 ^a^	↑ drug resistance	↓ cisplatin-induced apoptosis and regulates LASS2	[46]
miR-66-3b ^b^	↑ proliferation	↓ TUSC2, p53 and p21	[47]
miR-200a-3p ^a^	↑ invasion	↑ MMP-2 expression through Dicer/miR-16/JNK2/MMP-2 axis	[46]
miR-146-5p ^a^	↑ invasion	↑ ETS2-Mediated mmp2 mRNA transcription	[56]
**Suppressors of Oncogenesis**			
miR-205-5p ^a,^*	↑ apoptosis ↓ EMT and invasion	regulates the expression of the tumor-suppressor protein PTEN targets the transcriptional repressors of E-cadherin, ZEB1 and ZEB2	[11,46]
miR-132-3p ^a^	↑ angiogenesis, migration ↓ invasion and EMT	TGFβ1/Smad2 signaling pathway	[45]
miR-200c ^a^	↓ EMT, proliferation and invasion	↓ ZEB1/2 ↑ E-cadherin ↓ LDHA-induced glycolysis	[46]
miR-15a ^a^	↓ proliferation	Targets the oncogene BCL2	[46]
miR-30a-3p ^a^	↓ autophagy ↑ chemosensitivity to cisplatin ↓ invasion	↓ autophagy-related genes (including ATG5, ATG12, and Beclin-1) ↓ MMP-2 and MMP-9 expression	[46]
miR-503-5p ^a^	↓ proliferation	interferes with the Rb/E2F signaling pathway	[46]
Mirlet7b ^a,^**	↑ apoptosis ↓ drug resistance	targets MTDH, CALU and MTDH	[46]
miR-138-5p ^a^	↑ apoptosis	↓ Bcl-w and Akt1 protein expression	[56]
miR-144-5p ^a^	↓ proliferation	↓ cell cycle-related genes expression (CCNE1, CCNE2, CDC25A, and PKMYT1)	[56]
miR-145-5p ^a^	↓ proliferation and migration	Targets TAGLN2	[61]
miR-23b	↓ EMT induces G0/G1 cell cycle arrest and apoptosis	↓ expression of Zeb1	[61]
miR-133b ^b^	↑ apoptosis	↓ Bcl-w and Akt1 protein expression	[69]
miR-375-3p ^b^	↓ proliferation and invasion	↓ expression of FZD8 and therefore blocks the Wnt/β-catenin pathway	[73]
miR-29c ^b^	↓ proliferation	suppresses the G1/S cell cycle transition inhibits AKT and GSK-3β phosphorylation	[75]

**^a^**: measured in urine extracellular vesicles; ^b^: measured in blood extracellular vesicles; * mechanism described in various cancers; ** mechanism described in melanoma.

**Table 3:** Molecular pathways of EV RNA and LncRNA biomarkers in bladder cancer pathogenesis.

RNAs, lncRNAs Extracellular Vesicle Biomarkers in Bladder Cancer			
RNAs, lncRNAs	Effect	Mechanism of Action	Ref.
Promoters of Oncogenesis			
GALNT1 ^a^	maintenance of bladder cancer stem cells and bladder tumorigenesis	Mediates O-linked glycosylation of SHH to promote its activation	[44]
ARHGEF3 ^a,^*	↑ proliferation and invasion	↑ expression of Cyclin A2, Cyclin D1, and MMP2	[44]
UCA1 ^a^	↑ proliferation	regulates CREB	[53]
MALAT1 ^a^	↓ apoptosis	antagonizes miR-125b	[53,58]
UCA 201 ^a^	↑ migration and invasion	↑ the expression levels of ZEB1 and ZEB2 ↓ expression of hsa-miR-145 and its target gene, the actin-binding protein FSCN1	[53]
LINC 0035 ^a,^*	↑ proliferation, migration and invasion	↓ miR-466 and LYAR	[53]
SNHG16 ^b^	↑ proliferation, migration and invasion	activation of the Wnt/β-catenin pathway↑ expression of STAT3 by ↓ miR-98 expression↓ expression of Bax, cleaved-caspase-3 and cleaved-caspase-9	[54]
linc-UBC1 ^b^	↑ proliferation	binds to PRC2 complex and regulates target gene expression	[54]
PCAT-1 ^b,^**	↑ proliferation	↑ Myc and ↓ BRCA2	[54,58]
H19 ^b^	↑ proliferation, EMT and metastasis	↓ expression of E-cadherin↑ expression of ID2	[57]
**Suppressors of Oncogenesis**			
LASS2 ^a^	↓ cancer cell invasion and proliferation	↓ V-ATPase activity, the extracellular hydrogen ion concentration and, in turn, the activation of secreted MMP-2 and MMP-9	[44]
FOXO3 ^a^	↓ proliferation, migration and invasion	↑ miR-9-5p expression and thus ↓ TGFBR2 expression	[44]

^a^: measured in urine extracellular vesicles; ^b^: measured in blood extracellular vesicles; * mechanism described in lung carcinoma; ** mechanism described in various cancers.

## Data Availability

Not applicable.

## Abbreviations

BC: Bladder Cancer: SCC, Squamous Cell Carcinoma; EVs, Extracellular Vesicles; MVE, Multivesicular Endosome; ILVs, Intraluminal Vesicles; EMT, Epithelial to Mesenchymal Transition; CAFs, Cancer Associated Fibroblasts; HSPs, Heat Shock Proteins; NMIBC, non-muscle invasive bladder cancer; MIBC, muscle invasive bladder cancer; DUSP-1, Dual Specificity Phosphatase-1.
